# Physicochemical Properties and Cross-Cultural Preference for Mushrooms Enriched Third-Generation Potato Snacks

**DOI:** 10.3390/foods14234103

**Published:** 2025-11-28

**Authors:** Agnieszka Nemś, Maria Mora, Christina J. Birke Rune, Davide Giacalone, Luis Noguera Artiaga, Angel A. Carbonell-Barrachina, Joanna Kolniak-Ostek, Anna Michalska-Ciechanowska, Agnieszka Kita

**Affiliations:** 1 Department of Food Storage and Technology, Faculty of Biotechnology and Food Sciences, Wroclaw University of Environmental and Life Sciences, 51-630 Wrocław, Poland; 2 BCC Innovation, Technology Center in Gastronomy, Basque Culinary Center, 20009 Donostia-San Sebastián, Spain; mmora@bculinary.com; 3 Basque Culinary Center, Faculty of Gastronomic Sciences, Mondragon Unibertsitatea, 20009 Donostia-San Sebastián, Spain; 4 Department of Green Technology, University of Southern Denmark, DK-5230 Odense, Denmark; cjbr@iti.sdu.dk (C.J.B.R.); dg@iti.sdu.dk (D.G.); 5 SDU Climate Cluster, University of Southern Denmark, DK-5230 Odense, Denmark; 6 Instituto de Investigación e Innovación Agroalimentaria y Agroambiental (CIAGRO-UMH), Miguel Hernández University of Elche, 03312 Orihuela, Spain; lnoguera@umh.es (L.N.A.); angel.carbonell@umh.es (A.A.C.-B.); 7 Department of Fruit, Vegetable and Plant Nutraceutical Technology, Faculty of Biotechnology and Food Science, Wrocław University of Environmental and Life Sciences, 51-630 Wrocław, Poland; joanna.kolniak-ostek@upwr.edu.pl (J.K.-O.); anna.michalska@upwr.edu.pl (A.M.-C.)

**Keywords:** mushroom snacks, cross-cultural study, consumer preference

## Abstract

This study evaluated the physicochemical properties and sensory acceptance of third-generation, potato-based, extruded snacks enriched with mushroom powder (5% and 10%) across different European regions. The mushroom powder was produced from dried button mushrooms (*Agaricus bisporus*), ground and incorporated into the snack formulations, while control samples contained no mushroom powder. The investigation was conducted in Denmark, Poland, and Spain, involving 230 young adult consumers identified as the target demographic for this snack format. Incorporation of mushroom powder increased protein, fiber, ash, polyphenols, and antioxidant capacity while producing a darker color and crisper texture. Consumer acceptance varied across countries and was significantly influenced by individual differences in food involvement, neophobia, sustainability orientation, and dietary habits. Participants with greater health consciousness and sustainability engagement expressed a higher preference for snacks with greater levels of mushroom enrichment. Overall, moderate mushroom addition (5–10%) provides an optimal balance between enhanced nutritional quality, favorable sensory attributes, and broad consumer appeal, underscoring the potential of mushrooms as sustainable functional ingredients in snack product development.

## 1. Introduction

Across cultures, the perception and consumption of foods vary significantly, shaped by historical traditions, environmental factors, and socio-economic contexts. For instance, snacks are often associated with convenience and indulgence in Western societies, while in many Asian and Mediterranean countries, they can reflect traditional flavor profiles, local agricultural heritage, and symbolic [1]. Similarly, attitudes toward mushrooms are deeply culturally embedded: some societies display mycophilia, embracing mushrooms for culinary, medicinal, and folkloric purposes, while others exhibit mycophobia, avoiding fungi due to unfamiliarity or safety concerns [2].

Flavor preference is not purely biological—it is a learned cultural behavior. While humans are universally capable of perceiving the five basic tastes (sweet, sour, bitter, salty, and umami), the degree of liking and the foods people choose are strongly shaped by learning and culture [3]. Repeated exposure and familiarity (especially during childhood) increase liking and reduce neophobia. Cultural norms, symbolic meanings, and everyday food practices (what is customary, taboo, or prestigious) guide which tastes and dishes become preferred in a society. Social learning—modeling by parents, peers, and valued others—powerfully influences what children and adults accept and praise. Availability and the material/ritual context of foods (what’s locally produced, affordable, celebrated, or avoided) complete the picture: each nation’s traditional diet therefore functions like a “training system” for the palate [4]. Cross-cultural comparison of Poland, Spain, and Denmark reveals significant divergences in social organization, value orientation, and behavioral norms. According to Hofstede’s indexes for Poland, Spain, and Denmark, Polish culture is characterized by a relatively high power distance and uncertainty avoidance, reflecting an enduring respect for authority, structured hierarchy, and rule-based organization. In contrast, Spanish society demonstrates moderate power distance and a strong collectivist orientation, balancing hierarchical structures with interpersonal warmth, social cohesion, and cooperative interaction. Danish culture, rooted in egalitarianism and low power distance, promotes consensus-driven decision-making, informality, and open communication across social and professional contexts. These cultural distinctions are further reflected in lifestyle orientations: Poland exhibits greater restraint and traditionalism, Spain integrates discipline with sociability and pleasure, while Denmark prioritizes individual autonomy, social equality, and well-being. Collectively, these differences highlight how historical, social, and economic contexts shape distinct national patterns of interaction and life philosophy across Europe [5].

These culturally grounded food perceptions intersect with psychological dispositions, such as food neophobia—the reluctance to try unfamiliar foods—which varies across regions and influences openness to new or sustainable products [6]. This has direct implications for food choice, especially regarding sustainable alternatives such as plant-based proteins, insect-based snacks, or foraged mushrooms, which may be environmentally responsible yet culturally unfamiliar [7]. Even when environmental concern is high, sustainable eating habits are not universally adopted, with acceptance strongly mediated by cultural norms, sensory expectations, and prior exposure [8]. In this context, understanding the cross-cultural interplay between traditional food perceptions, neophobia, and sustainability-oriented consumption is essential. Such insights contribute to a better understanding of how cultural and psychological factors shape sustainable food choices and drive innovation [9].

Snack foods are popular among people of all ages. They are ready-to-eat products that do not replace main meals but are consumed between them to satisfy hunger or cravings. Snack foods have become increasingly popular due to convenience and changing lifestyles [10]. With the increasing number of people in society suffering from non-communicable diseases such as diabetes, hypertension, cancer, and heart disease, consumers pay more attention to the healthiness of products. In recent years, consumer preferences in the snack market have shifted from focusing mainly on taste and appearance to seeking high-quality products with balanced nutritional value [11].

Europe represents the world’s second-largest mushroom market, after the Asia–Pacific region. Leading producers include Poland, the Netherlands, and Spain [12]. Mushrooms, which are macrofungi from the Basidiomycota class, have characteristic fruiting bodies and have been used as a traditional dietary component for centuries. There are about 3000 species of edible mushrooms in the world, but only about 35 of them are commercially cultivated and accepted as food products [13]. The most popular species of these is the two-spore mushroom (Agaricus bisporus), followed by shiitake (Lentinula edodes) and oyster mushroom (Pleurotus eryngii) [14]. Mushrooms are low in fat and calories but rich in protein, dietary fiber, minerals, vitamins, and various bioactive compounds such as polyphenols and β-glucans, which contribute to their health-promoting properties [15,16,17,18]. Due to their excellent nutritional value and relatively low price, edible mushrooms are often called “poor man’s meat” [19]. However, mushrooms are appreciated not only for their nutritional value, but also for their characteristic aroma and taste [17]. The sensory attributes of mushrooms further contribute to their popularity in innovative food products.

Global mushroom consumption is growing at a rate of about 7% per year. According to FAO, global mushroom production has already exceeded 50 million tons per year [20]. However, fresh edible mushrooms such as button mushrooms are very perishable, which limits their long-term storage and transportation options. Because of their high water content and intense respiration rate, fresh mushrooms deteriorate rapidly through browning, softening, and nutrient loss. Among various preservation techniques, drying is considered one of the most effective, as it extends storage time and enhances product stability [21]. In the case of mushrooms, drying significantly prolongs their shelf life, allowing for longer storage and transportation without substantial quality degradation.

Dried mushrooms play an important role in the food industry, providing unique flavors and nutritional values. Their use includes the production of soups, sauces, ready meals, and spices. Mushrooms represent a promising alternative for innovation in the food industry, offering a unique combination of nutritional, functional, and sustainable benefits [22]. A new direction of using dried mushrooms can be applying them in recipes of popular snack products. However, it should be checked whether consumers are ready to accept such innovations.

This study investigates the physicochemical properties and cross-cultural consumer preferences of third-generation potato snacks enriched with mushroom powder. It is assumed that incorporating mushroom-derived ingredients can enhance the nutritional profile and introduce desirable umami notes, while simultaneously influencing expansion behavior, texture, and color due to changes in the starch matrix. Moreover, assume that consumer acceptance of mushroom-based products is known to vary across cultural contexts, largely reflecting differences in dietary habits, familiarity with mushroom flavors, and expectations toward snack products.

The aim of this study was to determine the physicochemical properties as well as the acceptance and perception of a new snack with the addition of different levels of dried mushrooms by different consumer groups in three European countries.

## 2. Materials and Methods

### 2.1. Product Characteristics

Fresh button mushrooms (*Agaricus bisporus*) were purchased from a local supermarket. Mushroom powder, used as an additive to the recipe of snacks, was obtained under laboratory conditions by drying sliced button mushrooms (*Agaricus bisporus*). The samples were put in a window with solar light heating on it within a great part of the day (pointing south) for 72 h. After drying, the slices were ground into a fine powder using a laboratory mill and stored in airtight plastic bags until further use. The material used for the study was samples of third-generation snacks based on potato products (grits, starch), corn flour, and salt with the addition of dried chamignons, *Agaricus bisporus* (mushroom powder) in the amount of 5% and 10%, expanded from extruded pellets in hot oil [23]. The control sample was snacks without the substitution of mushroom powder. Although the texture of the product was similar to some of the potato crisp snacks, its color and mushroom flavor could be considered atypical and new for most consumers.

### 2.2. Study Design

This study was divided into two parts: Part I determined the physicochemical properties of the analyzed snacks, and part II consisted of a cross-cultural consumer study (Table 1).

#### 2.2.1. Physicochemical Properties of Analyzed Snacks

##### Basic Chemical Composition

The dry matter, total protein, fat, ash, crude fiber, and salt contents were evaluated according to the methods of the Association of Analytical Chemists [24]. The dry matter content was determined on the basis of weight loss during thermal drying at 105 °C until a constant weight was achieved. Total nitrogen was determined by the Kjeldahl method using a Büchi Distillation Unit K-355 (Falwil, Switzerland). A nitrogen to protein conversion factor of 6.25 was used to calculate total protein, as a standard [25]. Fat content was determined using the Soxhlet method in a Büchi B-811 (Flawil, Switzerland) apparatus, with the use of diethyl ether as an eluent. The total ash content was determined by incinerating the sample in a muffle furnace at 550 °C and determining the weight of the residue. Total sugars were determined by a colorimetric method with 3.5 dinitrosalicylic acid (DNS) [26]. Data are reported as the mean value ± standard deviation (SD) for measurements.

##### Color

A Minolta CM-5 spectrophotometer (Konica-Minolta, Osaka, Japan) was used to evaluate the color of snacks. Color was expressed as lightness (L*), a* represents the green-to-red axis. Negative values indicate green, while positive values indicate red; b* represents the blue-to-yellow axis. Negative values indicate blue, while positive values indicate yellow, hue angle (*h*°), and chroma (*C*) color space parameters, and were measured against a white reference standard. Grounded snacks were used for color determination. Five measurements were taken on each sample, and the values for L*, a*, b*, *h*°, and *C* were averaged [27].

##### Texture

The texture of the obtained snacks was determined by an Instron type 5544 texture-measuring device with BlueHill software (version 4.13). The maximum force (N) necessary to break up a snack was measured using a shear blade at a displacement rate of 250 mm/min. Data are reported as the mean value of 20 measurements [27].

##### Antioxidant Capacity In Vitro

Antioxidant capacity tests were performed using a Synergy H1 microplate reader (BioTek, Winooski, VT, USA) based on the methodology described by Chandran et al. [28]. The antioxidant capacity was assessed using ABTS, FRAP, and DPPH assays and expressed as micromoles of Trolox equivalents per 1 g of snacks.

##### Polyphenol Profile

Polyphenol profiling was carried out using an ACQUITY UPLC system equipped with a photodiode array detector and a G2 Q-Tof mass spectrometer (Waters, Manchester, UK), according to the method described by Chandran et al. [28]. The resulting data were processed with MassLynx 4.0 ChromaLynx software (Waters). All analyses were carried out in triplicate (n = 3).

#### 2.2.2. Consumer Study General Procedure

The protocol and procedures used in this study were approved by the relevant institutional ethics committees (Mondragon Unibertsitatea, IEB20221115; Research Ethics Committee of the University of Southern Denmark, REC465; Miguel Hernandez University of Elche, PRL. DTA. ESN.01.22.; Wrocław University of Environmental and Life Sciences, N0N00000.0011.5.2024). All articles from the Declaration of Helsinki and the 2016/679 EU Regulation on the protection of natural persons regarding the processing of personal data and on the free movement of such data were met.

The experimental procedure was explained, and each participant gave consent indicating voluntary participation prior to beginning the study. The inclusion criteria were to be adults (above 18 years old) and able to provide informed consent, absence of non-communicable diseases, willingness to test new foods, and availability to participate in the study. A total of 238 young adult consumers aged between 18 and 34 years participated in the study. The youth segment was chosen because of the snack shape and texture of the designed product, since young consumers generally represent the core target segment for this type of snack product [29].

The study acceptance and perception of the new food, i.e., snacks with addition of mushrooms powder with 5 or 10% addition (as a control sample were snacks without addition of mushrooms powder), by different consumers groups and was developed in different countries: Denmark, representing Nordic countries (n = 79; mean age = 24.8 years old, 68% men), Poland, representing Central Europe, (n = 79; mean age = 22.6 years old, 27% men), and Spain, representing Mediterranean region (n = 80; mean age = 24.8 years old, 68% men).

Each participant received one snack of each type for evaluation (control, 5% mushrooms, and 10% mushrooms powder addition). The samples were labeled with three-digit codes and presented to the participants in a random order. Between the tasting of different samples, participants were provided with water and plain crackers to cleanse the palate and minimize carry-over effects. Participants received instructions to follow and test the samples of the new foods and a QR code that gave access to the questionnaire. The RedJade^®^ software v.5.1.1 (RedJade Sensory Solutions, LLC, Palo Alto, CA, USA) was used to collect responses. The tests were conducted in sensory labs/taste rooms under environmentally controlled conditions during March 2024.

#### 2.2.3. Consumer Test Questionnaires

The first part of the questionnaire included a question on liking (9-point hedonic scale, being 1 = dislike extremely, 5 = neither like nor dislike, and 9 = like extremely). After answering all questions linked to the product perception, a demographic questionnaire was completed by consumers. The questionnaire included questions on age, gender, the short version of the Food Choice Questionnaire [30], the Food Neophobia Scale [31], and questions on Dietary [32] and Sustainability [33] related habits. These questionnaires allowed understanding the main differences/similarities between the consumer groups as well as exploring the possibility of segmenting by attitudes and interests instead of only using consumer segments related to location. All questionnaires were translated and reviewed by native speakers of the different languages from the countries in which the study was performed (Spanish, Polish, and Danish).

### 2.3. Data Analysis

Differences in liking scores among the three snack formulations (control, 5% mushroom powder, and 10% mushroom powder) across the three countries (Spain, Poland, and Denmark) were examined using a two-way ANOVA, with Product and Country treated as fixed factors. Post hoc comparisons were performed using Tukey’s HSD test to identify significant pairwise differences.

To characterize participants’ cultural–psychological profiles, a Hierarchical Cluster Analysis (HCA) was performed on the complete dataset of each questionnaire—the Food Choice Questionnaire (FCQ), Food Neophobia Scale (FNS), and Sustainability Questionnaire (SQ)—including responses from all three countries. The analysis was conducted using Euclidean distance and Ward’s method of aggregation. Data were centered and standardized to eliminate scale effects. Subsequently, Fisher’s exact tests were applied to compare cluster distributions across countries, assessing differences in consumers’ responses to the FCQ, FNS, and SQ. Regarding Dietary Habits (DH), Fisher’s exact tests were also used on the different response categories to examine the distribution of dietary patterns across countries.

The physicochemical parameters of the three snack formulations—including proximate composition, color coordinates (L*, a*, b*), texture (fracture force), and antioxidant capacity (ABTS, DPPH, FRAP)—were analyzed using one-way ANOVA, followed by Tukey’s post hoc tests.

To identify the main drivers of product acceptance, relationships between the overall liking and the set of physicochemical and consumer variables were examined separately for each country. Pearson correlation analyses were performed to assess associations between liking scores, cultural–psychological factors, and physicochemical variables.

For all analyses, differences were considered significant at *p*-values < 0.05, unless otherwise stated. The statistical analyses were performed using XLSTAT (XLSTAT Version, 2021.5, Addinsoft, Paris, France).

## 3. Results and Discussion

### 3.1. Physicochemical Characterization of Snacks Used in the Experiment

The chemical composition and antioxidant capacity of the control sample and the snacks enriched with 5% and 10% mushrooms are presented in Table 2. The incorporation of mushrooms slightly increased the dry matter content, from 96.06% in the control to 97.07% in the snacks with 10% of mushrooms. A progressive rise in protein content was observed, ranging from 3.13% in the control to 4.56% in the 10% mushroom variant. Fat content increased substantially in the 5% mushroom sample (23.93%) compared to the control (17.78%), while the 10% sample showed a slightly lower value (22.80%). Ash content increased with mushroom addition, reaching 1.97% at the 10% level. The enrichment led to a reduction in total sugars, decreasing from 1.40% in the control to 0.61–0.66% in the mushroom-containing samples. Raw fiber content increased, with the highest value detected in the snacks with 5% of mushrooms (2.02%). Salt content remained relatively stable across all variants.

The observed chemical composition changes in the snacks enriched with mushrooms are consistent with prior studies of mushroom powder fortification in cereal-based products like pasta. The modest increase in dry matter as mushroom content rises aligns with reports that dried mushrooms contribute low moisture while supplying structural solids. The progressive rise in protein content (up to almost 1.5 times more in 10% mushroom snacks) is in line with findings in pasta and baked products fortified with dried mushroom powder, which showed significant protein enhancement [34]. Similarly, the increase in ash content reflects the mineral contribution of mushrooms, also documented in studies of mushroom-fortified pasta and biscuits [21]. The reduction in total sugars with mushroom addition reflects the lower carbohydrate (particularly simple sugars) content of mushrooms compared to many cereal or snack base materials, a trend also observed in fortified pasta where mushroom powder reduced digestible carbohydrates and lowered glycaemic response [34]. The increase in raw (dietary) fiber is given that mushrooms are a good source of insoluble and soluble fiber [16]. Meanwhile, the relatively stable salt content suggests that enrichment did not affect the formulation in terms of added sodium, which is favorable for maintaining sensory balance and health-related attributes. In conclusion, the results suggest that incorporating 5–10% dried mushrooms into snack formulations can beneficially alter the nutritional profile—particularly increasing protein, fiber, and ash contents.

Regarding bioactive compounds, mushroom addition caused a marked increase in flavanols (from 0.75 to 8.45 mg/kg), phenolic acids (from 26.25 to 126.30 mg/kg), and total polyphenols (from 26.99 to 135.75 mg/kg). Total polyphenols rose almost three-fold, from 6.92 mg/g in the control to 20.35 mg/g in the 10% mushroom snacks. Consistently, the antioxidant capacity measured by ABTS, DPPH, and FRAP assays increased significantly with mushroom addition. ABTS activity rose from 0.49 µmol Trolox/g in the control to 1.53 µmol Trolox/g in the 10% sample, while DPPH and FRAP values also followed the same upward trend. These findings indicate that mushroom enrichment substantially improves the nutritional profile and antioxidant potential of the product.

Regarding bioactive compounds, mushroom supplementation resulted in a marked increase in flavanols (up to 4.3 times more in snacks with 5% of mushrooms powder and 12 times more in snacks with 10% of mushrooms compared to control sample), phenolic acids (up to 3 times more in snacks with 5% of mushrooms powder and 4.8 times more in snacks with 10% of mushrooms compared to control sample), and total polyphenols (respectively, 3 and 5 times in 5% and 10% mushrooms snacks compared to control sample).

Beyond macronutrient modification, mushroom addition markedly increased the levels of bioactive compounds, particularly flavanols, phenolic acids, and total polyphenols. These compounds are well-documented contributors to the antioxidant potential of edible mushrooms [35]. In this study, the nearly threefold increase in total polyphenols in the 10% enriched snacks was accompanied by a significant rise in antioxidant capacity across ABTS, DPPH, and FRAP assays, demonstrating that phenolic enrichment translates directly into functional benefits. Similar correlations between polyphenol content and radical-scavenging capacity have been reported for both mushroom extracts and mushroom-fortified bakery products [35,36]. These findings reinforce the potential of mushrooms not only as a source of essential nutrients but also as natural antioxidants capable of enhancing product functionality.

The incorporation of mushroom powder induced significant modifications in the color characteristics of the samples (Table 3). Lightness (L*) exhibited a progressive reduction, with values declining by more than one-third at the highest level of mushroom addition, indicating a markedly darker appearance. Lightness (L*) decreased markedly from 85.58 in the control to 63.40 and 55.73 in samples with 5% and 10% mushroom addition, respectively. Redness (a*) increased substantially, rising over five-fold relative to the control, thereby confirming a clear shift toward red tones. The a* value (redness) increased progressively from 0.96 in the control to 4.17 and 5.28, confirming a shift toward red tones. In contrast, yellowness (b*) displayed only a minor reduction (less than 5%), suggesting that this attribute was not markedly influenced by mushroom incorporation. Chroma (*C*) values showed no pronounced variations across treatments (19.24–19.57), indicating that overall color saturation was maintained. The hue angle (*h*°) decreased from 87.18° in the control to 77.47° and 74.36° in the mushroom-enriched samples, reflecting a noticeable shift in hue from yellow toward red with increasing levels of mushroom powder. The incorporation of mushroom powder produced pronounced shifts in color parameters, in line with prior reports of mushroom powder’s influence on the visual appearance of cereal and snack products. The marked decline in lightness (L*) reflects the inherently darker pigments from mushroom powder [37]. The concomitant increase in redness (a*) with increasing mushroom content also mirrors findings in baked goods where darker mushroom powders shift color toward red/brown hues, with limited change in yellowness (b*) [38]. The relatively stable chroma values suggest that although hue and brightness changed, the saturation or color purity remained roughly consistent, indicating that the visual intensity of color was preserved despite hue shifts.

Texture analysis further revealed that hardness was significantly affected by mushroom addition. At the highest inclusion level, hardness declined by more than one-third compared to the control, demonstrating that mushroom powder incorporation contributes to the development of a more crispy product texture. It suggests that mushroom powder contributes to a more brittle or crisp texture. This is consistent with some previous work, where mushroom supplementation decreased the hardness of products, likely due to interference with starch–protein matrix continuity and altered water binding [39]. In summary, the color and texture changes both carry important implications for consumer acceptance: a darker, redder appearance may be perceived as more “natural” or “earthy,” but excessive darkening may reduce appeal.

### 3.2. Acceptability of the Snacks Used in the Experiment

A consumer acceptance analysis of the mushroom snacks was conducted to evaluate how the perceived acceptability of these snacks varied depending on the consumer’s country of origin, the level of addition of mushroom powder, as well as the interaction between these factors. This approach allowed for the identification of both cultural differences in preferences and product-specific effects, while also highlighting how the combination of consumer background and product characteristics jointly influenced overall acceptance.

Mushrooms possess a complex and distinctive flavor profile dominated by umami, often described as a deep, savory, and slightly earthy taste [40]. Poland and Spain both exhibit a high cultural tolerance and liking for umami, yet through distinct sociocultural and dietary mechanisms. In Poland, umami familiarity is rooted in nature-based culinary traditions, especially mushroom foraging and cooking. Regular childhood exposure to these flavors promotes sensory learning and reduces food neophobia, a relationship well established in developmental taste research [41]. Mushroom picking functions as a family and cultural ritual, strongly associated with heritage and emotional bonding, reinforcing positive affective associations with the savory, earthy taste [42]. Thus, umami in Poland is domestic and nostalgic, emerging from local ecology and tradition rather than foreign influence. In Spain, umami is embedded in the Mediterranean dietary pattern, rich in tomatoes, seafood, aged cheeses, and dry-cured ham (jamón ibérico), all abundant in free glutamates and nucleotides [43]. Culinary practices such as sofrito and slow cooking amplify these compounds, producing synergistic umami intensity. Spanish cuisine also relies on social and hedonic reinforcement—shared meals, tapas, and leisurely dining—which link umami flavors to pleasure, conviviality, and satisfaction [44]. In contrast, Denmark displays a more recent, innovation-driven relationship with umami. The rise in the New Nordic Cuisine movement introduced fermentation, seaweed, and mushroom-based preparations, increasing awareness and appreciation of savory flavors [45]. However, this remains a culinary movement rather than a deeply rooted national taste tradition.

#### Liking of the Samples

**Figure 1:**
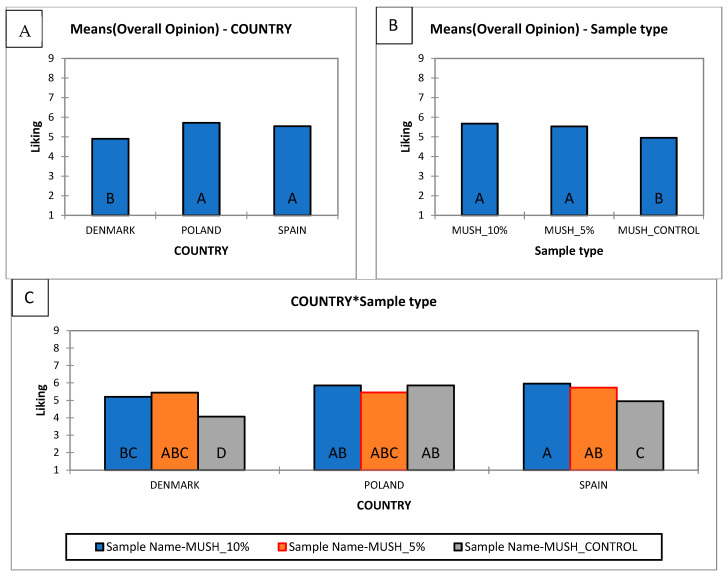
Liking of the snacks of the control sample and with the addition of 5% and 10% of mushroom powder by country (**A**), kind of snacks (**B**), interaction country and type of snacks (**C**). Figure 1A,B show the main effects from two-way ANOVA, Figure 1C: two-way interaction effect (Sample X Country). A, B, C, D—different letters in the rows statistically significant differences between means following Tukey’s post hoc tests; (*p* < 0.05).

During this study, consumers rated their “liking” for the snacks given to them for evaluation. The ANOVA revealed significant effects of both country and sample type on overall opinion, as well as clear interactions. Concerning the country factor, Polish and Spanish consumers rated the samples significantly higher than Danish consumers (Figure 1A). With respect to the sample factor, mushroom-enriched formulations (5% and 10%) were preferred over the control across all countries (Figure 1B). The country-by-sample interaction indicated that preferences differed among countries: Danish and Spanish consumers clearly preferred the mushroom-enriched sample over the control, whereas Polish consumers perceived no significant differences (Figure 1C).

The cross-country sensory evaluation demonstrated that mushroom powder enrichment (5–10%) increased the overall liking of snacks in Denmark and Spain, whereas in Poland, all variants were rated similarly. This pattern is consistent with previous research showing that mushroom-derived compounds enhance flavor intensity, particularly through umami-related taste attributes, and simultaneously contribute to a healthier product image [46]. The lack of differentiation in Poland may reflect cultural familiarity with mushroom flavors, where long-standing culinary traditions contribute to consistently high acceptance regardless of enrichment level [47]. It is also possible that a ceiling effect occurred, as baseline liking of all snack variants was already high. Together, these findings highlight that consumer responses to mushroom enrichment are shaped not only by sensory enhancement but also by cultural context, which should be carefully considered in product development and cross-market positioning.

Although mushrooms are increasingly recognized as a health-promoting food and are being incorporated into innovative food products, consumer acceptance remains a barrier to their widespread adoption, particularly in Western markets where mushrooms are not a traditional dietary staple [48]. The analysis of consumer preferences across three countries (Denmark, Poland, and Spain) revealed notable differences in the evaluation of mushroom-enriched snacks.

Clear differences emerged between countries’ liking of the control sample snacks. Danish consumers are showing no strong preferences for any of the mushroom snack variants. Polish consumers displayed a relatively neutral stance, with no extreme tendencies in their assessments. By contrast, Spanish consumers demonstrated more pronounced preferences, showing greater openness to novel flavors and particularly favoring samples containing a higher proportion of mushrooms (notably 10%).

From a market perspective, a moderate addition of mushrooms has the best chance to succeed in all three countries. The positioning of Polish and Spanish consumers near the centroid indicates alignment with the overall preference trend, which corresponds with previous findings reporting greater openness of these populations toward products with stronger flavors and umami characteristics [49].

In summary, mushroom enrichment improves the nutritional profile of extruded snacks, while sensory acceptance tends to increase with higher levels of substitution. A 5% and 10% incorporation levels appear to offer the best balance between functional benefits and consumer satisfaction. These results highlight the importance of integrating both nutritional enhancement and sensory optimization in the development of mushroom-based functional foods.

Polish consumers place substantial value on traditional products and familiar flavors, which helps explain their heightened emphasis on product familiarity. For many people, food should be safe, known, and predictable. Research into Poland’s food market confirms that traditional and regional food attributes—including origin, quality, and historic connection—play an increasingly important role in consumer choice [50]. In contrast, consumers in Spain exhibit greater openness to culinary experimentation and novel flavors, rendering “product familiarity” a less critical factor for them. This aligns with Spain’s rich Mediterranean culinary heritage, which is characterized by diverse regional ingredients and dynamic flavor combinations [51].

### 3.3. Consumer Clusters

The consumers who participated in the study were divided into clusters based on a set of lifestyle and dietary-related factors. The key criteria included their food choices, level of food neophobia, sustainability habits, and overall dietary habits. This approach made it possible to identify groups of consumers with similar attitudes and behaviors, allowing for a better understanding of differences in preferences and enabling the development of tailored nutritional and communication strategies for each cluster.

#### 3.3.1. Food Choice

**Figure 2:**
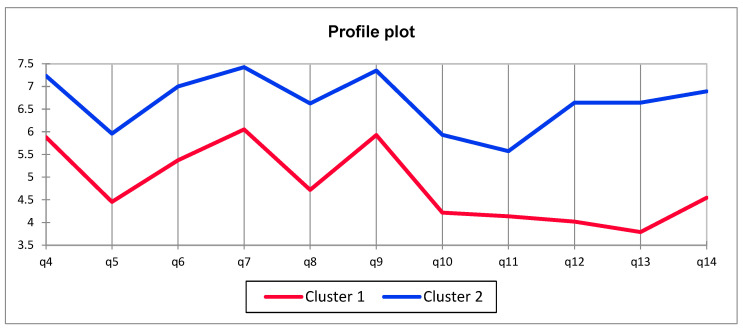
Comparison of Cluster 1 and Cluster 2 responses to the Food Choice Questionnaire.

Consumers who took part in the research were very diverse. In terms of food choice based on answers to questions from the food choice questionnaire, consumers can be divided into two clusters (Figure 2). In general, consumers are grouped into those who place more importance on all aspects of food choice and those who place less importance on all aspects of food choice. For cluster 1FCQ, which was significantly more present among Polish consumers (65,4%) than Spaniards (24,7%), the most important things are that the food is healthy (q4), provides pleasure (q7), and is affordable (q9). And the least important thing if it is animal and environmentally friendly or not (q12 and q13). For cluster 2FCQ, which is more present in Spain (75.3%) than in Poland (34.6%), the most important things were that the food is healthy (q4), provides pleasure (q7), and is affordable (q9). And the least important thing if it is familiar or not (q11). Polish consumers gave less importance to animal and environmentally friendly aspects of food; moreover, Spaniards gave less importance to familiarity with the product (Table 4).

#### 3.3.2. Food Neophobia

**Figure 3:**
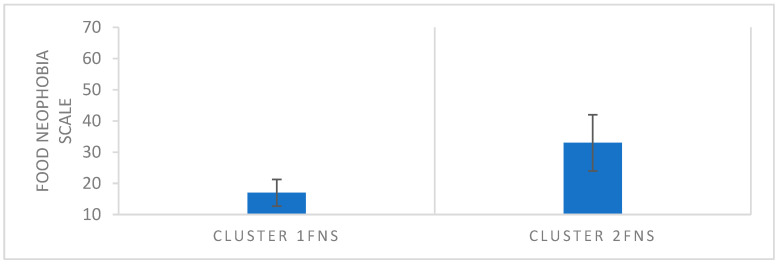
Comparison of Cluster 1FNS and Cluster 2FNS Responses to the Food Neophobia Scale.

Analyzing results from the neophobia scale, consumers can be divided into two groups: Cluster 1FNS- neophilic, which are willing to try new or unknown products, and Cluster 2FNS -neophobic, which are afraid to try new foods (Figure 3). The question “I am very particular about the food I will eat” is the reason for rejection in both groups. Consumers in all three countries are very particular about the food they will eat. Cluster 1FNS (neophilic) is significantly more present among Polish and Danish consumers (respectively, 42.3% and 44.3%) than Spaniards (21.9%). Cluster 2 (neophobic) is significantly more present in Spain (78.1%) than in Poland (57.7% and Denmark (55.7%). According to data presented by Jeżewska-Zychowicz et al. [52], in Poland, 14.4% of the population exhibits food neophobia, 12.2% can be classified as food neophilic, while the majority maintain a neutral attitude toward novel foods (Table 5).

#### 3.3.3. Sustainability Questionnaire

**Figure 4:**
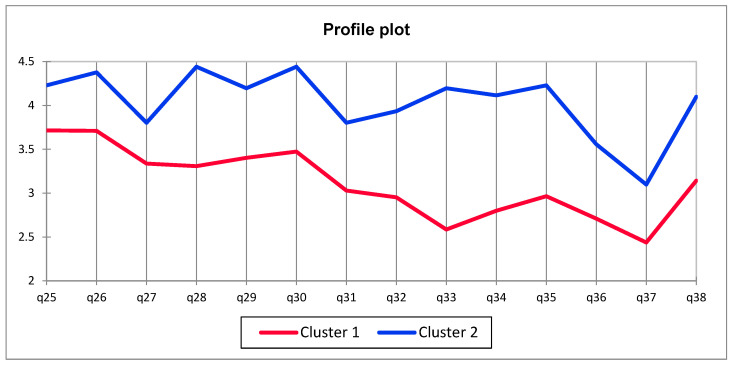
Comparison of Cluster 1 and Cluster 2 responses to the Sustainability Questionnaire.

Results from the Sustainability Questionnaire showed that consumers can be divided into two clusters: Cluster 1SQ, with less engagement or involvement in environmental actions, and Cluster 2SQ—more engaged with environmental actions (Figure 4). Generally, it was observed that cluster 1SQ was more present in all countries in comparison to Cluster 2SQ, regardless of the country. Moreover was observed that consumers from Denmark were less engaged with environmental actions than in Poland or in Spain (Table 6). According to the European Commission, in Poland, only around 40% of individuals engage in climate-related actions on a daily basis, although the majority believe they have an impact on the environment. Denmark stands out with the highest level of public support for combating climate change (69%), and more than half of its citizens have been actively involved in such efforts for over 50% of the time in recent months. In Spain, both environmental awareness and the sense of personal responsibility are very high, with participation in pro-environmental activities exceeding half of the population [53].

The incorporation of mushrooms into the production of new snack products offers a promising approach, as mushrooms are considered a raw material with a low environmental footprint. They require less land, water, and energy, and can be cultivated on agricultural by-products such as straw and sawdust, thereby contributing to waste reduction and resource efficiency [54]. In contrast to many alternative protein sources, mushrooms are generally more sustainable, demanding fewer resources and producing lower greenhouse gas emissions [55]. As sustainability becomes a critical consideration for both consumers and producers, mushrooms present an eco-friendly option that aligns with the principles of the circular economy [56].

#### 3.3.4. Dietary Habits

The results from the dietary habits questionnaire showed that consumers can be divided into five clusters (Table 7). Cluster 1DH represented consumers who consciously limit meat and meat products but allow their consumption (flexitarians). Cluster 2DH represented omnivore consumers who eat everything. Cluster 3DH describes semi-vegetarians who do not eat red meat but eat fish, poultry, and shellfish. Cluster 4 DH represents consumers who are half-vegetarians, like pescatarians or lactoovovegetarians. Cluster 5DH were vegans. Cluster 2DH (omnivores) was the most frequent group in all countries and had an average of 49.1%. Whereas the Cluster 1DH was similar in all countries and stated 27.4%. Differences were observed in Cluster 3DH (semi-vegetarianism), where in Poland it was 21.8%, while in Denmark it was only 5.1%. According to data published by World Population Review in 2025, vegans account for 1.8% of the population in Poland, 4% in Denmark, and only 0.8% in Spain. The proportion of vegetarians is lowest in Spain (1.4%), while in Poland and Denmark, vegetarians represent 8.4% and 10% of the population, respectively [57]. Data presented by the organization ProVeg International indicate that flexitarians constitute 24% of the Polish population and approximately 30% of the Spanish population [58].

### 3.4. Correlation Between Liking of the Snacks and All Consumers and Products Variables Run per Country

No robust linear associations were observed between overall liking and the set of physicochemical or attitudinal variables. Correlations with liking were small (≤ ~0.35). Taken together, these patterns suggest that, for all the countries, product formulation (at 0/5/10% mushroom) and country-level context are more informative than individual difference measures for explaining acceptance.

## 4. Conclusions

The enrichment of extruded third-generation snacks with 5% and 10% mushroom powder significantly improved their nutritional and functional properties without compromising overall consumer acceptance. The addition of mushrooms increased protein, fiber, ash, and bioactive compound contents—particularly flavanols, phenolic acids, and total polyphenols—leading to a marked enhancement in antioxidant capacity. These improvements demonstrate that mushrooms are an effective natural ingredient for increasing the nutritional and functional quality of cereal-based snacks.

Mushroom supplementation also influenced the snacks’ physical characteristics, resulting in darker, redder coloration and reduced hardness, contributing to a crispier texture. Such changes may enhance the perception of naturalness, although excessive darkening could affect visual appeal.

Consumer testing conducted in Denmark, Poland, and Spain revealed that acceptance of mushroom-enriched snacks depends both on formulation and cultural background. Danish and Spanish consumers preferred enriched variants, whereas Polish consumers rated all samples similarly—possibly due to greater familiarity with mushroom flavors. Cluster analyses indicated that food involvement, neophobia, sustainability engagement, and dietary habits influenced preferences. Health-conscious and sustainability-oriented consumers were more open to higher mushroom levels, while neophobic individuals favored the control.

Overall, moderate mushroom addition (5–10%) provides an optimal balance between enhanced nutritional value, appealing sensory properties, and broad consumer acceptance across diverse European markets. The findings underscore the potential of mushrooms as sustainable, functional ingredients that can improve both the health profile and marketability of novel snack products.

The findings of this study may have practical relevance for European snack manufacturers, particularly those engaged in the production of potato-based products. The integration of mushroom-derived ingredients offers opportunities to diversify product portfolios and enhance sensory appeal through the addition of natural umami and earthy flavor notes. Moreover, the incorporation of mushrooms can contribute to an improved nutritional profile of snack products, providing additional sources of dietary fiber, protein, vitamins, minerals, and bioactive compounds with antioxidant properties. Such innovations align with contemporary consumer demands for healthier, more authentic, and functionally enriched snack options within the European market.

## Figures and Tables

**Table 1:** The details regarding the measurement of variables in the study.

Physico-Chemical Properties		Consumer Study
Basic chemical composition	Dry matter [%]	Acceptability of the snacks (9-pt hedonic scale)
	Total proteins [%]	Food choice (Appendix A)
	Fat [%]	Food neophobia (Appendix B)
	Ash [%]	Sustainability Questionnaire (Appendix C)
	Total sugars [%]	Dietary habits (Appendix D)
	Raw fiber [%]	
	Salt [%]	
Bioactive compounds	Total flavanols HPLC [mg/kg]	
	Total phenolic acids HPLC [mg/kg]	
	Total polyphenols HPLC [mg/kg]	
Antioxidant capacity	TEAC ABTS [µmol TE/g]	
	TEAC DPPH [µmol TE/g]	
	TEAC FRAP [µmol TE/g]	
Color parameters	L*	
	a*	
	b*	
	*C*	
	*h°*	
Texture [N]		

**Table 2:** Chemical composition and antioxidant capacity of snacks with the addition of dried mushrooms expanded by frying in hot oil.

Parameter	Control Sample	Mushrooms 5%	Mushrooms 10%
Dry matter [%]	96.06 ± 0.11 c	96.50 ± 0.08 b	97.07 ± 0.08 a
Total proteins [%]	3.13 ± 0.10 c	3.95 ± 0.08 b	4.56 ± 0.08 a
Fat [%]	17.78 ± 0.16 c	23.93 ± 0.08 a	22.80 ± 0.17 b
Ash [%]	1.13 ± 0.11 c	1.70 ± 0.05 b	1.97 ± 0.11 a
Total sugars [%]	1.40 ± 0.03 a	0.61 ± 0.11 b	0.66 ± 0.04 b
Raw fiber [%]	1.33 ± 0.16 c	1.86 ± 0.11 b	2.02 ± 0.07 a
Salt [%]	1.40 ± 0.12 a	1.55 ± 0.08 a	1.40 ± 0.13 a
Total flavanols HPLC [mg/kg]	0.75 ± 0.01 c	3.23 ± 0.02 b	8.45 ± 0.04 a
Total phenolic acids HPLC [mg/kg]	26.25 ± 0.33 c	77.32 ± 0.99 b	126.30 ± 0.95 a
Total polyphenols HPLC [mg/kg]	26.99 ± 0.34 c	81.02 ± 1.03 b	135.75 ± 0.94 a
TEAC ABTS [µmol TE/g]	0.49 ± 0.01 c	1.01 ± 0.00 b	1.53 ± 0.01 a
TEAC DPPH [µmol TE/g]	0.45 ± 0.03 c	0.64 ± 0.03 b	0.72 ± 0.04 a
TEAC FRAP [µmol TE/g]	0.24 ± 0.01 c	0.74 ± 0.02 b	1.21 ± 0.04 a

a, b, c—different letters in the rows indicate statistically significant differences (one-way ANOVA; Tukey’s post hoc tests; *p* < 0.05, n = 3).

**Table 3:** The color and texture of the snacks, with the addition of mushrooms, were expanded by frying in hot oil.

Parameter	Control Sample	Mushrooms 5%	Mushrooms 10%
L*	85.58 ± 0.26 a	63.40 ± 0.48 b	55.73 ± 0.37 c
a*	0.96 ± 0.11 c	4.17 ± 0.04 b	5.28 ± 0.04 a
b*	19.51 ± 0.24 a	18.78 ± 0.09 a	18.84 ± 0.15 a
*C*	19.54 ± 0.25 a	19.24 ± 0.10 a	19.57 ± 0.15 a
*h*°	87.18 ± 0.33 a	77.47 ± 0.08 b	74.36 ± 0.10 c
Texture [N]	33.34 ± 5.59 a	22.34 ± 5.42 b	21.13 ± 5.15 b

a, b, c—different letters in the rows indicate statistically significant differences (one-way ANOVA; Tukey’s post hoc tests; *p* < 0.05; color n = 5; texture n = 20).

**Table 4:** Percentage cluster share and *p*-values from Fisher’s exact test comparing the distribution of clusters between countries (Poland, Denmark, and Spain) based on results from the Food Choice Questionnaire. Statistically significant results (*p* < 0.05) are shown in red with “*”.

Cluster	Cluster Description	Cluster Share			*p*-Value		
		Poland	Denmark	Spain	Poland	Denmark	Spain
		%					
Cluster 1FCQ	Consumers who place more importance on all aspects of food choice	65.4	51.9	24.7	0.000 *	0.406	<0.0001 *
Cluster 2FCQ	Consumers who place less importance on all aspects of food choice	34.6	48.1	75.3	0.000 *	0.406	<0.0001 *

Values displayed in red with “*”are significant at the level alpha = 0.05.

**Table 5:** Percentage cluster share and *p*-values from Fisher’s exact test comparing the distribution of clusters between countries (Poland, Denmark, and Spain) based on results from the Food Neophobia Scale. Statistically significant results (*p* < 0.05) are shown in red with “*”.

Cluster	Cluster Description	Cluster Share			*p*-Value		
		Poland	Denmark	Spain	Poland	Denmark	Spain
		%					
Cluster 1FNS	Neophilic consumers	42.3	44.3	21.9	0.197	0.085	0.002 *
Cluster 2FNS	Neophobic consumers	57.7	53.7	78.1	0.197	0.085	0.002 *

Values displayed in red with “*”are significant at the level alpha = 0.05.

**Table 6:** Percentage cluster share and *p*-values from Fisher’s exact test comparing the distribution of clusters between countries (Poland, Denmark, and Spain) based on results from the Sustainability Questionnaire. Statistically significant results (*p* < 0.05) are shown in red with “*”.

Cluster	Cluster Description	Cluster share			*p*-Value		
		Poland	Denmark	Spain	Poland	Denmark	Spain
		%					
Cluster 1SQ	Consumers are less engaged with environmental actions.	69.2	83.5	67.1	0.344	0.012 *	0.150
Cluster 2SQ	Consumers are more engaged with environmental actions	30.8	16.5	32.9	0.344	0.012 *	0.150

Values displayed in red with “*”are significant at the level alpha = 0.05.

**Table 7:** Percentage cluster share and *p*-values from Fisher’s exact test comparing the distribution of clusters between countries (Poland, Denmark, Spain) based on results from the Dietary habits questionnaire. Statistically significant results (*p* < 0.05) are shown in red with “*”.

Cluster	Cluster Description	Cluster Share			*p*-Value		
		Poland	Denmark	Spain	Poland	Denmark	Spain
		%					
Cluster 1DH	Consumers who limit meat and meat products in their diet (flexitarians)	25.6	29.1	27.4	0.755	0.756	1.000
Cluster 2DH	Consumers who are eating everything (omnivores)	41.0	54.4	52.0	0.095	0.268	0.573
Cluster 3DH	Consumers who do not eat red meat but eat fish, poultry, and shellfish (semi-vegetarians)	21.8	5.1	12.3	0.007 *	0.012 *	1.000
Cluster 4DH	Half-vegetarians like pescatarians or lactoovovegetarians	10.3	8.9	6.9	0.623	1.000	0.619
Cluster 5DH	Vegans	1.3	2.5	1.4	1.000	0.609	1.000

Values displayed in red with “*”are significant at the level alpha = 0.05.

## Data Availability

The data supporting the findings of this study are available on request from the corresponding author.

## Appendix A

**Table A1:** Food choice questionnaire. Please read the following statements carefully, and select the response that you consider best reflects your usual behavior using the scale below, It is important to me that the food I eat on a typical day is…:

Type of Questionnaire	Question Number	Behavior	Scale
Food choice questionnaire (FCQ)	Q4	Healthy	1 = Not at all important 2= 3= 4 = Neither/nor 5= 6= 7 = Very important
	Q5	A way of monitoring my mood	
	Q6	Convenient	
	Q7	Provides me with pleasurable sensations	
	Q8	Natural	
	Q9	Affordable	
	Q10	Helps me control my weight	
	Q11	Familiar	
	Q12	Environmentally friendly	
	Q13	Animal friendly	
	Q14	Fairly traded	

## Appendix B

**Table A2:** Food neophobia scale. How much do you agree or disagree with the following statements:.

Type of Questionnaire	Question Number	Statement	Scale
Food Neophobia Scale (FNS)	Q15	**“I am constantly sampling new and different foods”** *Reversed item*	1 = Strongly disagree 2= 3= 4 = Neither agree/nor disagree 5= 6= 7 = Strongly agree
	Q16	“I don’t trust new food”	
	Q17	“If I don’t know what is in a food, I won’t try it”	
	Q18	**“I like food form different cultures.”** *Reversed item*	
	Q19	“Food from other cultures looks too weird to eat.”	
	Q20	**“At social gatherings, I will try a new food”** *Reversed item*	
	Q21	“I am afraid to eat food I have never had before”	
	Q22	“I am very particular about the food I will eat”	
	Q23	**“I will eat almost anything”** *Reversed item*	
	Q24	**“I like going to places serving foods from cultures different to my own”** *Reversed item*	

## Appendix C

**Table A3:** Sustainability Questionnaire. Read the following statements and rate how important this is to you:.

	Question Number	Statement	Scale
Sustainability Questionnaire (SQ)	Q25	Recycling	1 = I am not aware of this issue/I have never paid attention to it 2 = I am aware of this issue but I am not interested in it 3 = I am aware of this issue, I am interested in it, but I haven’t done anything for it 4 = I am interested in this issue and I have done something for it 5 = I am interested in this issue and I have taken a significant action
	Q26	Respect of the environment	
	Q27	Enhancing renewable/alternative energies	
	Q28	Enhancement of local production	
	Q29	Preserving natural resources	
	Q30	Supporting local economy	
	Q31	Enhancement of free-preservatives food products	
	Q32	Enhancing sustainable production labels (i.e., ecolabels)	
	Q33	Enhancement of non-industrial farming	
	Q34	Enhancement of organic cultivation	
	Q35	Promoting environmental sustainable production	
	Q36	Contrasting the new developing countries exploitation	
	Q37	Contrasting GMO’s food	
	Q38	Promoting workers’ rights	

## Appendix D

**Table A4:** Dietary habits. Please choose ONE of the following statements that best represent your dietary habits:.

	Number of Question	Options
Dietary habits (DH)	Q39	1 = I regularly eat red meat, fish and chicken
		2 = I consciously reduce meat intake, but eating meat now and then
		3 = I don’t eat red meat, but I eat fish, chicken and other poultry
		4 = I don’t eat red meat nor chicken, but I eat fish and shellfish
		5 = I don’t eat meat or fish, but I eat eggs and dairy products
		6 = I don’t eat meat, fish or eggs, but I eat dairy products
		7 = I don’t eat meat, fish or dairy products, but I eat eggs
		8 = I don’t eat meat and I don’t use products of animal origin
		9 = I eat organic and locally grown foods, with a great overlap with foods consumed in a vegetarian diet, yet also including some kinds of meat
